# Functional characterization of nutraceuticals using spectral clustering: Centrality of caveolae-mediated endocytosis for management of nitric oxide and vitamin D deficiencies and atherosclerosis

**DOI:** 10.3389/fnut.2022.885364

**Published:** 2022-08-15

**Authors:** Anton Franz Fliri, Shama Kajiji

**Affiliations:** Emergent System Analytics LLC, Clinton, CT, United States

**Keywords:** atherosclerosis, cardio miracle, caveolae-mediated endocytosis, endothelial cells dysfunction, nitric oxide, TGF beta degradation, spectral clustering of protein swarms, vitamin D efficacy

## Abstract

It is well recognized that redox imbalance, nitric oxide (NO), and vitamin D deficiencies increase risk of cardiovascular, metabolic, and infectious diseases. However, clinical studies assessing efficacy of NO and vitamin D supplementation have failed to produce unambiguous efficacy outcomes suggesting that the understanding of the pharmacologies involved is incomplete. This raises the need for using systems pharmacology tools to better understand cause-effect relationships at biological systems levels. We describe the use of spectral clustering methodology to analyze protein network interactions affected by a complex nutraceutical, Cardio Miracle (CM), that contains arginine, citrulline, vitamin D, and antioxidants. This examination revealed that *interactions between protein networks affected by these substances modulate functions of a network of protein complexes regulating* caveolae-mediated endocytosis (CME), TGF beta activity, vitamin D efficacy and host defense systems. Identification of this regulatory scheme and the working of embedded reciprocal feedback loops has significant implications for treatment of vitamin D deficiencies, atherosclerosis, metabolic and infectious diseases such as COVID-19.

## Introduction

The term “nutraceuticals” includes dietary supplements, functional foods, vitamins, and nutritional products. These products are generally mixtures of natural products, vitamins, minerals and/or herbal ingredients. For the most part, clinical evidence is generally limited at the ingredient level since in the United States it is optional to make claims of clinical benefit to bring a nutraceutical to market. Further, unlike pharmaceuticals, nutraceuticals offer narrow profit margins; this in-conjunction with the non-stringent global regulatory environment allows manufacturers to avoid running expensive and time-consuming clinical trials for demonstrating health benefits for gaining marketing approval. However, with a plethora of products in the marketplace, it is becoming more and more important to competitively position the nutraceutical in terms of its health and wellness benefits.

Critical for predicting health effects of the nutraceuticals is the understanding of how complex mixtures of substances (ingredients, probes) influence the propagation of information in biological networks (1). While the structures and functions of cellular components are relatively well understood, very little is known on how system components work or cease to work together in case of injuries, medications or diets (1). This knowledge gap can largely be attributed to the complexity of network-network interactions giving rise to system plasticity and emergent properties (2). Thus, system perturbations affecting behavior frequently display “modularity” and “interdependence” wherein modularity refers to effects on system components that by working together deliver well defined outputs, and interdependence refers to effects of the perturbations on the organization of components necessary for delivering optimal end results (3). In the framework of network biology, interdependence results from interactions between networks of tissues, cells and proteins (4). However, predicting properties regulated by interacting network systems in real-world settings requires large amounts of data and, if absent, escapes the reach of even the most sophisticated statistical methodologies (3). For addressing this gap, applications of various spectral clustering methodologies have been explored (6, 7).

We have developed a novel spectral clustering methodology for advancing these efforts. It allows tracking of perturbation-induced information flows through multiple interacting network systems and facilitates determination of cause-effect relationships for even complex mixtures (see section “Materials and methods”).

The aim of this study was to use spectral clustering for determining cause-effect relationships of the nutraceutical, Cardio Miracle (CM) marketed as a nitric oxide booster (see Supplementary Data 1), containing amongst its 50 + ingredients arginine, citrulline, vitamin D and antioxidants, which have recently been shown to increase the bioavailability of NO and decrease oxidative stress *in vitro* (8). Previous studies have linked vitamin D and NO deficiencies to nutrient-sensing (10, 11). It has also been shown that the addition of antioxidants to a combination of arginine, citrulline and vitamin D synergistically increases the ratio between NO and peroxynitrite production in endothelial cells (9). Critical for endothelial cell function; these signaling systems are important for health: endothelial cells dysfunction (ED) plays a key role in development of cardiovascular diseases, diabetes, obesity, and inflammatory conditions (12).

We describe the use of spectral clustering for identifying how CM affects the propagation of signals and impacts biological processes. We present evidence that interaction(s) between arginine, citrulline, vitamin D3 and antioxidants not only synergistically balance NO and peroxynitrate generation, but also affect functions of a protein network that regulates caveolae mediated endocytosis (CME), TGF beta activity, and vitamin D efficacy. Identification of this regulatory scheme and the working of embedded reciprocal feedback loops advances our understanding of how the various signaling systems/biological processes interact at a body-wide scale and generates meaningful hypotheses for bridging the gap between preclinical and clinical studies.

## Materials and methods

Cardio miracle, marketed by Evolution Nutraceuticals (12), is a NO supplement. It is a mixture of arginine, citrulline (13), cholecalciferol vitamin D, various vitamins, quercetin, minerals and over 700 natural products that can be isolated from herbal and vegetable product constituents (see Supplementary Material Section) (13–15).^1^

Spectral Clustering Methodology, specifically designed to discover emergent properties resulting from network-network interactions, has been described in detail in PCT/US2016/06379. Its application for analysis of CM is detailed in the work-flow below.


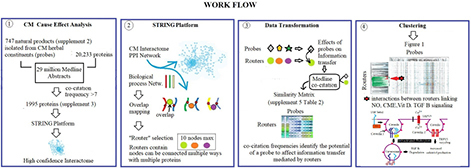


**FIGURE 1:**
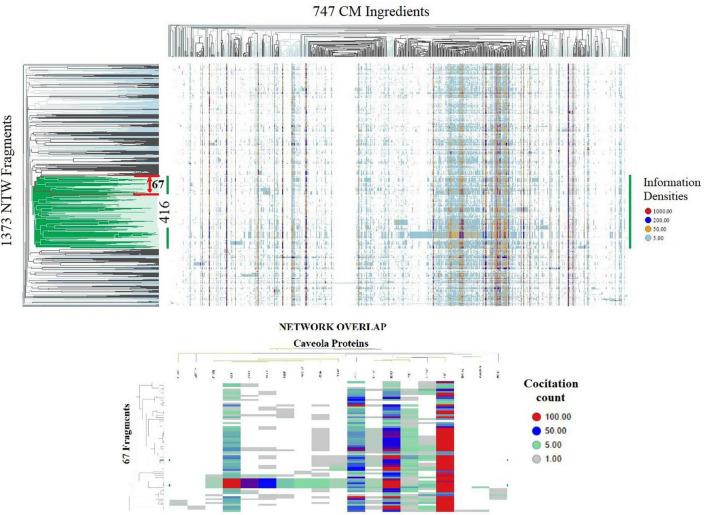
Spotfire generated heatmaps. TOP shows on the vertical dendrogram axis the organization of 1,373 CM interactome fragments overlapping with biological process network and identifies network-network interactions; the horizontal dendrogram axis identifies phenotypes of 747 natural products and CM ingredients containing substance groups inducing similar interactions in network fragment phenotypes; the vertical dendrogram section highlighted in green identifies network-network connectivity of core group of 416 network fragments induced by all 747 CM ingredients. BOTTOM identifies that a phenotype of 67 Fragment within the red boundary of the top heatmap containing biological process regulating vitamin D metabolism, Nitric oxide production and redox balance overlap with a network regulating caveola functions. Shown are the information densities of proteins CAV1, CAV3, PTRF, CAV2, FLOT1, FLOT2, NOS3, SRC, STOM, PRKCDBP, EHD2, SLC6A3, KIF18A, PACSIN2, CDH1, ADTRP, TFPI, PTGIS, PLVA with 67 network fragments.

Specifically,

(1) Protein network components affected by CM ingredients were identified by determining co-occurrence frequencies of 700 natural products isolated from its herbal constituents with 20,233 proteins in over 17 million Medline abstracts. This data-gathering step resulted in the selection of 1,995 proteins with a co-occurrence frequency count of more than seven.

(2) Use of the STRING platform’s highest confidence in network connectivity level for protein network construction delineated a high confidence CM interactome (see Supplementary Material Section) (2). STRING’s gene enrichment analysis using biological process networks as background was used to divide the 1,995 protein-containing CM interaction network into smaller network fragments. This step resulted in > 4,000 protein network fragments that overlap with biological process networks regulating functions throughout the body. Selection of network fragments with < 10 network nodes (associated with a strength of > 0.9 and a *p* value of < 0.0001) produced 1,373 biological structure-function constraint network fragments.

(3) These 1,373 network fragments were used as topological descriptors for determining information densities of 747 CM ingredients associated with these 1,373 fragments in the Medline database resulting in the generation of a similarity matrix containing 1,373 × 747 information density measurements (Supplementary Material Section: Supplementary Table 2).

(4) Clustering of this similarity matrix using the TIBCO Spotfire platform (15)^2^, and cosine correlation as similarity measure provided Figure 1.

## Results

Identification of molecular underpinnings of the observation that interaction(s) between arginine, citrulline, vitamin D3 and antioxidants synergistically balance NO and peroxynitrate generation, using hierarchical clustering of the 1,373 × 747 similarity matrix in SPOTFIRE revealed that 747 CM ingredients induce network-network interactions involving a core group of 416 network fragments (Figure 1).

Using STRING’s highest confidence level for investigating the connectivity between these 416 network fragments identified networks of protein complexes containing 1,320 proteins overlapping with networks involved in the regulation of caveolae-associated functions, redox stress (17), and nutrient sensing. Of relevance to the regulation of endothelial functions is that the flattening of the curvature of caveolae under mechanical pressure (e.g., shear stress, blood pressure) that functions as a stress sensor (18) and modulates CME (18, 19). CME, by connecting mechanical input signals to the nucleus, regulates system-wide responses to ED associated with NO and vitamin D deficiency (20–24).

**FIGURE 2:**
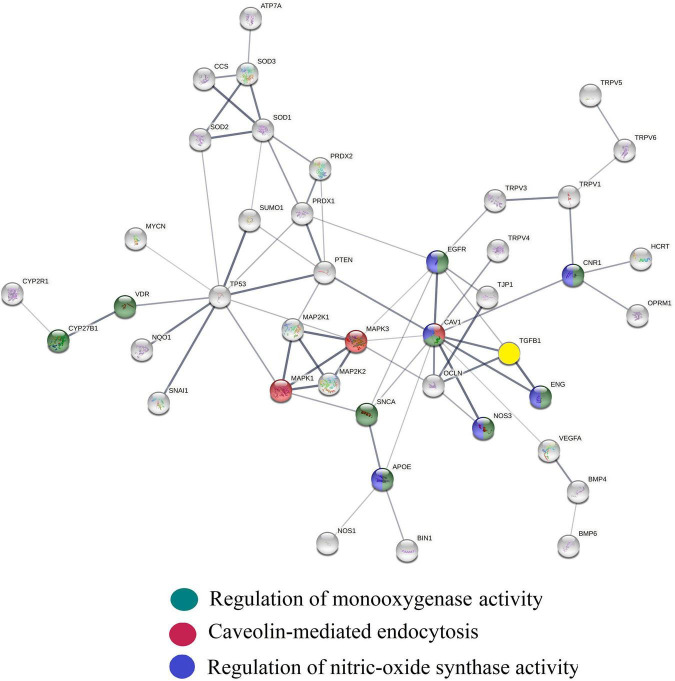
Protein interaction network generated using the String platform’s highest confidence level (0.9) and CM interactome proteins: ADCYAP1, ADRB1, ADRBK1, AKAP5, AKT1, APOE, ATF4, ATP7A, BIN1, BMP2, BMP4, BMP6, CACNA1C, CACNA1D, CASQ2, caveolin1, CCK, CCS, CD320, CD9, CEACAM1, CNR1, CUBN, CYBA, CYP19A1, CYP1A1, CYP1A2, CYP1B1, CYP24A1, CYP26A1, CYP27B1, CYP2R1, CYP3A4, CYP3A4, CYP7A1, ENG, ENO1, EREG, F2, FGF19, FGF23, FGFR1, FOXO1, FOXO3, HCRT, HMOX1, IFNG, IL1B, IL4, ISCU, JAG1, KLF4, LEP, LMNA, MAPK3, MPO, MTOR, NF1, NFATC1, NFKB1, NOS1, NOS3, NOTCH1, NPY, NQO1, OCLN, OPRM1, ORAI1, PARK7, PDGFRA, PGR, PIAS4, PINK1, PLTP, POR, PPARA, PPARGC1A, PRDX1, PRDX2, PRKCD, PTEN, PTGS2, RBPJ, RORA, SCARB1, SIRT1, SMAD3, SNAI1, SNCA, SOD1, SOD2, SOD3, SPP1, STAT3, STIM1, STRA6, SUMO1, TGFB1, TJP1, TLR2, TNF, TNFRSF1A, TREM2, TRPV4, UBIAD1, UCN, UGT1A1, UGT1A8, VDR, VEGFA. Edges show physical interactions between proteins. Biological processes overlapping with this network fragment regulate caveola mediated endocytosis (yellow), NO biosynthesis (green), vitamin D activation (red) and TGF beta activity (light blue). Caveolin 1 (caveolin 1) serves as a hub protein connecting these biological process networks.

Determination of the connectivity between CM interactome fragments overlapping with biological process networks involved in NO and vitamin D signaling (Figures 1, 2) identified caveolin 1 (caveolin 1), a major structural component of caveolae, as a key regulator of endothelial nitric oxide synthetase (NOS3) activity, vitamin D activation (VDR/CYP27B1), TGF beta activity and CME. Evidence discussed below supports the premise that this functional relationship represents a novel mechanism for regulating vitamin D efficacy.

## Discussion

Spectral clustering based functional analysis of CM provides valuable insights regarding supplement-mediated regulation of vitamin D in cardiovascular diseases and for exploring ED. CM delivers vitamin D in form of pure cholecalciferol and as a mixture of cholecalciferol and 25-hydroxycholecalciferol (25(OH)D3) in the form of shitake and maitake mushroom powders. Cholecalciferol and 25-hydroxycholecalciferol are precursors of the hormone 1,25 dihydroxyvitamin D3 (calcitriol) and reach the bloodstream via intestinal absorption. The oral bioavailability of vitamin D precursors is limited by the ABCA1 transport protein which functions as an intestinal absorption barrier (25–27). Determination of co-investigation frequencies of 747 CM ingredients and ABCA1 transport protein was used to establish that the natural product, quercetin (28) and catechins (found in abundance in CM’s antioxidant ingredient group) can inhibit ABCA1 transport and thereby, increase the oral bioavailability of vitamin D (29). Clinical observations provide strong evidence that co-administration of quercetin with vitamin D increases the oral bioavailability and efficacy of vitamin D and support the premise that CM ingredients enhance the oral bioavailability of cholecalciferol vitamin D (30, 31).

**FIGURE 3:**
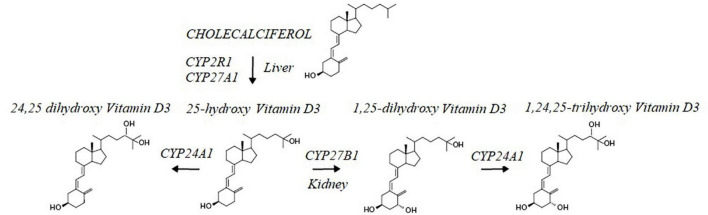
Cholecalciferol Vit D is metabolized in the liver by CYP2R1 and CYP27a1 into the Vitamin D3 precursor, 25 hydroxy Vitamin D3. This intermediate is further metabolized by CYP24A1 into a hormonal inactive form of Vit D3; 24,25 dihydroxy vitamin D3 and in the kidney by CYP27B1 into the active from of Vit D3, 1,25 dihydroxy-Vitamin D3. Levels of the hormonal active form of Vit D3 are decreased through the action of CYP24A1 converting the hormone into 1,25,25-trihydroxy Vitamin D3.

As shown in Figure 3, cytochrome CYP2R1 and CYP27A1 convert cholecalciferol into 25-hydroxyvitamin D (32) and which, upon binding to vitamin D binding protein and albumin (33, 34) circulates in the bloodstream. Vitamin D binding protein regulates circulating free and total levels of vitamin D metabolites, where ∼0.03% of 25(OH)D is free and 99.97% is bound to the vitamin D binding protein and to albumin. Cytochrome CYP24A1 transforms 25 (OH)D3 into an inactive metabolite 24,25-di-hydroxyvitamin D. Activation of 25 (OH)D3 bound to vitamin D binding protein requires active transport via megalin-mediated endocytosis into kidney proximal tubule cells and conversion by CYP27B1 into calcitriol. Calcitriol is metabolized by cytochrome CYP24A1 into its inactive form, 1,24,25 (OH)3 vitamin D (35) and has a half-life of ∼ 6 h vs. the half-life of the inactive form (25 (OH)D3) which is up to 3 weeks (36). Considering the plethora of vitamin D effects, the balance between vitamin D inactivating and activating metabolic enzymes and the speed with which calcitriol and its precursors can enter cells determines overall efficacy profile of vitamin D – and is therefore, dynamically regulated (37).

Amongst the many vitamin D efficacy regulators is calcitriol itself; it adjusts the expressions of CYP27B1, CYP24A1 and the vitamin D receptor (VDR) (38, 39). Calcitriol upon binding to VDR (enriched in caveolae) (40) is transported via CME across cell membranes (41). This caveolae-mediated active transport of receptor bound vitamin D3 is activated by NO (42) and inhibited by peroxynitrite (43, 44). Since CM has been documented to increase levels of bioavailable NO and lower peroxynitrate concentrations, it has therefore the capacity to activate and stabilize of CME leading to increased cellular uptake and genomic activity of vitamin D3 (41, 45–47).

The premise that CME activation increases calcitriol production is grounded in observations that CME regulates activity of a vanillin-type selective calcium channel TRPV5, present on the apical membrane of distal kidney tubule epithelial cells (48, 49), and that the loss of TRPV5 channel activity causes calcitriol overproduction and vitamin D hypervitaminosis (50, 51). Hence CME activation, by removing TRPV5 from the cell surface, decreases TRPV5 activity and increases calcitriol production (52, 53). This fine-tuning of CME-mediated vitamin D activation involves protein kinase C (PKC) and phospholipase D (PLD) wherein the activation of PKC inhibits CME and the inhibition of PKC activates CME (54). Since vitamin D3 (55) in combination with PKC inhibitors quercetin (56), oleanolic acid (57, 58), and curcumin (59) in CM’s herbal constituents reinforces CME activation, this ingredient combination is projected to increase calcitriol production. The vitamin D efficacy of CM is further enhanced by oleanic acid, a natural product isolated from CM’s hawthorn and mango extracts, which decreases the expression and protein levels of the calcitriol inactivator CYP24A1 (60).

**FIGURE 4:**
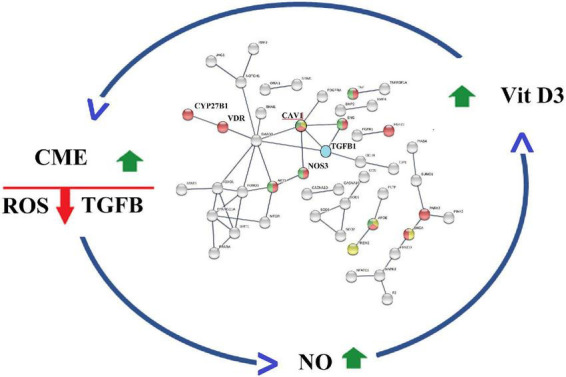
Caveola mediated endocytosis (CME) modulates activities of a reciprocal feedback loops that finetunes ROS production, TGF beta activity, Nitric oxide levels O and Calcitriol production.

Activation of CME also increases the degradation of TGF beta (61), an immunosuppressive cytokine that upregulates ROS production (62), arginases expression, and decreases NO production (63, 64). Thus, CM-mediated activation of CME is expected to reduce TGF beta signaling; this inhibitory effect is enhanced by hesperidin, a natural product isolated from CM’s citrus extracts, which downregulates TGF beta expression (65). CME-mediated decrease in TGF beta activity adds to CM’s projected capacity to increase vitamin D efficacy (66). This is because TGF beta has been shown to inhibit the expression of megalin, the intracellular protein essential for uptake of 25 hydroxyvitamin D by kidney proximal tubule cells and its subsequent conversion to calcitriol by CYP27B1 (67, 68). Accordingly, the experimental observations summarized in Figure 4, provides strong support for the existence of a CME-based regulatory scheme that upon activation controls functions of reciprocal feedback loops that decrease ROS, decrease TGF beta activity, and increase NO and calcitriol production (45).

Inhibition of TGF beta signaling has been shown to impede progression of atherosclerosis and results in regression of established disease (69). Thus, CM-mediated activation of CME in combination with hesperidin’s capacity to downregulate TGF beta expression is projected to have beneficial effects in atherosclerosis. Secondly, CM-mediated increase in calcitriol production via CME-activation is projected to lower caveolin 1 level. This is therefore expected to also intercept development of atherosclerosis since increased caveolin 1 expression is linked to disease progression (70–73). The mechanistic rationale is that caveolin 1 levels are regulated by autophagy and prevent its own degradation since it is itself an autophagy inhibitor (64, 74, 75). Caveolin 1-mediated inhibition of autophagy is reduced by calcitriol which activates tyrosine kinase activity of pp60src and results in the phosphorylation of caveolin 1 at tyrosine 14 and increased autophagy (76–78).

Phosphorylation of caveolin 1 at tyrosine 14 also increases endothelial nitric oxide synthase activity and increases NO production to further reinforce the CM feedback loop. Thus, the capacity of CM to enhance TGF beta and caveolin 1 degradation is projected to enhance the anti-atherosclerotic efficacy of this nutraceutical (79–82).

It is also important to note that CM’s projected pharmacology to lower caveolin 1 level by increasing autophagic degradation is enhanced by other autophagy-activating CM ingredients: palmitic acid (83), resveratrol (84), pterostilbene (85), quercetin (86), piceatannol (87), delphinidin (88), cyanidin-3-o-glucoside (89), and sulforaphane (89).

## Summary

Identification of network-network interactions regulating reciprocal feedback loops advances our understanding of how ingredients/natural products of nutraceuticals interact within a system and provide guidance for product optimization and improving preclinical and clinical outcomes. Spectral clustering of protein interaction information associated with ingredients of a complex nutraceutical supplement, CM, uncovered several biological functions supported by its ability to correct cellular redox imbalance. These include its ability to increase oral bioavailability of cholecalciferol vitamin D3 and to activate and stabilize caveolin-mediated endocytosis. The combination of these functionalities infers involvement of reciprocal cellular feedback loops that increase NO production and vitamin D3 efficacy, decrease TGF beta signaling and oxidative stress, and activate autophagy. Since down-regulation of TGF beta activity and activation of autophagy is anticipated to intercept/reverse endothelial dysfunction associated diseases including atherosclerosis (90–93), diabetic kidney disease (94–96), and COVID-19 (97–99), supplementation with CM-like functionalities is projected to benefit treatment of these diseases. For validating these effect predictions, clinical trials are warranted.

## Data availability statement

The original contributions presented in the study are included in the article/Supplementary material, further inquiries can be directed to the corresponding author.

## Author contributions

AF developed the spectral clustering methodology and performed the data analysis of this study. SK contributed to the interpretation of analysis outcomes. Both authors contributed to the manuscript and agreed to be accountable for the content of the work.

## Footnotes

1
https://www.cardiomiracle.com/

2
https://www.tibco.com/products/tibco-spotfire
