# Supplementary material for: Functional characterization of nutraceuticals using spectral clustering: Centrality of caveolae-mediated endocytosis for management of nitric oxide and vitamin D deficiencies and atherosclerosis
Source: Front Nutr. 2022 Aug 15;9:885364. doi: 10.3389/fnut.2022.885364 (PMC9421303; doi:10.3389/fnut.2022.885364)
Supplement: Supplementary file 1 [file Data_Sheet_1.docx]

Supplementary Material

# Supplementary Data: Cardio Miracle Product Ingredients

L-Citrulline, L-Arginine, L-Arginine Alpha Ketoglutarate, Agmatine Sulfate, Beet Powder, Acacia Senegal, Sea Sal, Carrot Powder, Coconut Powder, D-Ribose, Spinach, Broccoli, Carrot, Sweet Potato, Orange, Apple, Strawberry, Sunflower Seeds, Shiitake Mushroom, Maitake Mushroom, Turmeric Powder, Hawthorne Fruit Extract, Grape Seed Extract, Pine Bark Extract, L-Ornithine HCL, L-Theanine, Acerola Cherry Juice Powder, Blueberry Powder, Taurine, Quercetin Dihydrate, AstraGin (Astragalus Membranaceus and Panax Notoginseng),. Vitamin D-3 Cholecalciferol, Alpha Lipoic Acid, Cranberry Fruit Extract, Magnesium L-Threonate, Pomegranate fruit extract, Vitamin E, Mixed Tocopherols, Zinc Amino Acid Chelate, Zinc Oxide, Astaxanthin Powder, Co Enzyme Q10, OmniMin AC Trace Mineral, Black Pepper extract, Piperine, Vitamin K2, Selenium Amino acid chelate, Vitamin B12, Biotin, Erythritol, Citric Acid, DL-Malic Acid, Raspberry flavor, Natural blackberry lemonade flavor, Silica, Stevia.

CM, core ingredients are the combination of arginine, citrulline and cholecalciferol Vit D. By supplying arginine CM increases plasma arginine levels shown to provide the fuel for nitric oxide synthase (NOS) mediated NO generation;^^[[1]](#endnote-1)^^ Vit D; in turn; is known to enhance NO generation by increasing the expression of eNOS and decreasing arginase activities which reduce the bioavailability of arginine and cause eNOS uncoupling and endothelial dysfunction.^^[[2]](#endnote-2)^;^[[3]](#endnote-3)^^  The addition of folate; calcium; magnesium; and potassium to the product supports endothelial nitric oxide synthetase (eNOS) activity.^^[[4]](#endnote-4)^^

# Natural products isolated from CM herbal constituents

(E)-2-hexenal, (S)-Carvone, (Z)-3-hexenal, (Z)-3-Hexenyl acetate, (Z)-beta-Ocimene, 1,2,3,4,6-Penta-O-galloyl-beta-D-glucose, 1,4-Cineole, 1,8-Cineole, 11-Eicosenoic acid, 1-Methoxyspirobrassinin, 1-Octanol, 1-O-trans-cinnamoyl-beta-D-glucopyranose, 2,4,5-Trimethoxybenzaldehyde, 2,6-Dimethoxy-1,4-benzoquinone, 20(R)-Ginsenoside Rg3, 20(R)-Ginsenoside Rh2, 28-Norcastasterone, 2alpha,3beta-Dihydroxyolean-12-en-28-oic acid, 2-Aminoethanol, 2-Carene, 2-C-Methyl-D-erythritol 4-phosphate, 2-Methylheptane, 2-Pentadecanone, 2-Phenylethyl alcohol, 2-Undecanone, 3,3'-di-O-Methylellagic acid, 3,5-Dicaffeoylquinic acid, 3-Carene, 3-Hydroxyanthranilic acid, 3-O-Caffeoylquinic acid, 3-O-Galloylquinic acid, 3-Pentanol, 3-Phenylpropanol, 4-(4-Hydroxyphenyl)-2-butanone, 4,5-Dicaffeoylquinic acid, 4-coumaric acid, 4-hydroxy-2,5-dimethyl-3(2H)-furanone, 4-hydroxybenzoic acid, 4-Hydroxyphenylpyruvate, 4-Hydroxyproline, 5,7,4'-Trihydroxy-3'-methoxyflavone, 5-Deoxystrigol, 5-Hydroxytryptamine, 5-Methoxytryptamine, 5-Oxoproline, 6-Methyl-5-hepten-2-one, 7-O-methylisomucronulatol, abietic acid, Abscisic acid, Acacia senegal, Acetic acid, adenin, adenosine, Adipic acid, Adrenaline, Aesculetin, Aesculin, agmatine sulfate, alanin, alpha tocopherol, alpha-Asarone, alpha-Bisabolol, alpha-carotene, alpha-Eleostearic acid, alpha-Farnesene, alpha-Guaiene, alpha-Ketoglutaric acid, alpha-lipoic acid, alpha-phellandrene, alpha-Pinene, alpha-Terpinene, alpha-Terpineol, alpha-Tocopherol, alpha-Tocopherolquinone, alpha-Tocopherylquinone, alpha-Tocotrienol, Ampelopsin, Amurensin, Ananas comosus, andrographolide, Antheraxanthin, anthocyanidins, anthocyanins, apigenin, Apigenin-7-O-glucoside, Arabinogalactan, Arachidic acid, arachidonic acid, Arbutin, Arjuna, Aromadendrene, ar-Turmerone, Ascaridole, Ascorbic acid, Asiatic acid, aspartic acid, Astragalin, Astragaloside I, Astragaloside II, Astragaloside III, Astragaloside IV, Astragalosides II, Astragalosides IV, astringin, a-tocopherol, Austroinulin, Avicularin, Ayapin, Bacillus coagulans, behenic acid, Benzeneacetaldehyde, Benzeneboronic acid, Benzoic acid, Benzylglucosinolate, bergamottin, Bergaptol, beta carotene, Beta vulgaris, Beta-1,6-glucan, beta-Alanine, beta-carotene, beta-Caryophyllene, beta-cryptoxanthin, beta-Elemene, beta-Eleostearic acid, beta-Eudesmol, Betalains, beta-Myrcene, Betanidin, Betanin, beta-Pinene, beta-sitosterol, beta-Sitosterol glucoside, beta-Tocopherol, Betavulgarin, Betulinic acid, Bicyclogermacrene, Biochanin A, Biotin, bisdemethoxycurcumin, borneol, Brassica oleracea, Brassicanal C, Brassinin, Brassinolide, Brevifolin, Brevifolincarboxylic acid, bromelain, Butin, caffeic acid, Caffeine, caffeoylquinic acid, Calamenene, calcium, Calebin A, Calenduloside E, Campesterol, camphene, Camphor, capric acid, Caproic acid, Caprolactam, capsaicin, Carbanilide, Carene, carotenes, Carotol, Carrot anthocyanin, carvacrol, caryophyllene, caryophyllene oxide, Castalagin, Castasterone, Casuariin, Casuarinin, catalase, Catechin, Cellobiose, chlorogenic acid, Chlorophyll a, Chlorophyll b, cholecalciferol, Cholesterol, Cholesterol sulfate, choline, Chrysoeriol, cineol, Cinnamate, Cinnamic acid, Cinnamtannin B1, Cinnamtannin D1, cis-3-Hexen-1-ol, cis-p-Coumaric acid, cis-Rose oxide, Citral, Citric acid, Citronellol, citropten, Citrulline, citrullus lanatus, citrus aurantium, Citrus limon, Cladrin, cobalamin, Cochliophilin A, Cocos nucifera, Coniferyl alcohol, Copaene, Copalyl diphosphate, Copper, Corilagin, Cosmosiin, Costunolide, coumaperine, Coumarin, Coumestrol, Cryptochlorogenic acid, Cubenol, Cucumopine, Cucurbitacin I, cuparene, curcuma longa, Curcumenol, Curcumenone, curcumin, Curdione, Cyamopsis tetragonoloba, cyanidin, Cyanidin 3,5-di-O-glucoside, Cyanidin 3-arabinoside, cyanidin 3-glucoside, cyanidin 3-rutinoside, cyanidin-3-glucoside, Cyanidin-3-O-glucoside, Cyanin, Cyclo-DOPA, cynarin, Cynaroside, Daidzein, Daidzin, Decanal, Dehydroabietic acid, Dehydroascorbate, Dehydrocurdione, Delphin, delphinidin, Delphinidin 3-O-glucoside, delphinidin-3-glucoside, delta-Cadinene, delta-Carotene, delta-Elemene, delta-Tocopherol, delta-Tocotrienol, demethoxycurcumin, dencichine, dextrin, D-Galactose, D-Galacturonic acid, D-Glyceric acid, diacetyl curcumin, Dihydrocaffeic acid, dihydrochalcones, dihydroferulic acid, dihydrokaempferol, Dihydroxyphenylalanine, Dihydrozeatin, dimethylglycine, Diosgenin, diosmetin, diosmin, D-Mannose, D-Mannose-1-phosphate, DMAPP, Dopamine, Dopaquinone, D-Ribose, D-Xylose, Elemicin, ellagic acid, ellagitannins, ent-Kaur-16-en-19-oic acid, ent-Kaurene, Epicatechin, epicatechin gallate, epicatechins, Epigallocatechin, ergocalciferol, ergosterol, ergosterol peroxide, ergothioneine, eriocitrin, Eriodictyol, eritadenine, erythritol, Estradiol, Ethanol, Ethyl crotonate, Ethyl decanoate, Ethyl propionate, eugenol, Eupatin, Eupatolide, Eupatorin, Euxanthone, Fagopyritol B2, falcarindiol, Falcarinol, Ferulenol, ferulic acid, Feruloylputrescine, Feruloylquinic acid, Fisetin, flavanols, flavones, flavonoids, flavonols, Folate, Folic acid, Formononetin, fragaria, Fructose, Fucosterol, Fumaric acid, GABA, Gadoleic acid, Galactinol, galacturonic acid, Gallagic acid, gallic acid, Gallocatechin, gamma-Aminobutyric acid, gamma-Carotene, Gamma-Linolenic acid, gamma-Nonalactone, gamma-Terpinene, gamma-Tocopherol, gamma-Tocotrienol, GDP-L-galactose, Gemin D, Genistein, Gentisic acid, Geranylgeranyl diphosphate, Geranyllinalool, Germacrene D, germacrone, Gibberellin A1, Gibberellin A3, Gibberellin A4, Ginsenoside F1, Ginsenoside F2, Ginsenoside Ra3, ginsenoside Rb1, Ginsenoside Rb2, Ginsenoside Rb3, Ginsenoside Rc, Ginsenoside Rd, ginsenoside Re, Ginsenoside Rf, Ginsenoside Rg1, Ginsenoside Rg2, Ginsenoside Rg3, Ginsenoside Rh1, Glucobrassicanapin, Glucobrassicin, Glucocheirolin, Glucoerucin, Glucogallin, Glucoiberin, Gluconapin, Glucoputranjivin, glucoraphanin, Glucose, Glucuronate, glucuronic acid, glutamic acid, glutaric acid, glutathione dehydrogenase, glutathione reductase, Glyceraldehyde 3-phosphate, glycine, Glycocholic acid, Glycolic acid, Granatin A, Granatin B, Grifola frondosa, guaiacol peroxidase, Guaijaverin, Gypenoside XVII, helianthus anuus, Helicid, Heptanal, Herbacetin, hesperetin, hesperidin, Hexahydroxydiphenic acid, Hexan-1-ol, Hexanal, Histamine, homocitric acid, homoorientin, Hordenine, Hovetrichoside C, humulene, hyperoside, Idaein, Imperatorin, Indole, Indole-3-acetaldehyde, indole-3-acetic acid, Indole-3-acetonitrile, indole-3-carbinol, Indole-3-carboxylic acid, Indole-3-ethanol, Indoleacetic acid, Inosine, Inositol, Ipomoea batatas, IPP, iron, Isoamyl acetate, Isoamyl alcohol, Isoastragaloside II, Isobetanin, Isoborneol, isocitric acid, Isocorilagin, Isoeugenol, isoflavone, Isolariciresinol, isoleucine, Isomangiferin, Isopelletierine, Isophorone, Isopimaric acid, Isopimpinellin, Isoprene, Isoprocurcumenol, Isorhamnetin, Isoschaftoside, Isovaleraldehyde, Isovitexin, Jasmonic acid, kaempferol, Kinetin, L-alanine, Laminaribiose, Lanosterol, L-Arginine, L-asparagine, L-aspartic acid, lauric acid, L-Citrulline, lentinan, Lentinula edodes, Leptosin, leucine, Leucopelargonidin, L-Galactono-1,4-lactone, L-Galactose, L-Glutamic acid, L-Gulono-1,4-lactone, L-Gulose, L-Histidine, L-Homocysteine, lignoceric acid, limocitrin, Limonene, limonin, linalool, Linalyl acetate, linoleic acid, linolenic acid, Linolic acid, lipoic acid, Lirioresinol B, L-Malic acid, L-Methionine, Loliolide, L-Ornithine, L-phenylalanine, L-Theanine, L-Threonic acid, L-Threonic acid magnesium salt, L-tryptophan, L-tyrosine, lutein, Luteolin, L-valine, lycopene, Lyoniside, lysine, magnesium, Maitake, malic acid, Malonic acid, Malonyl-CoA, Malonyl-ginsenoside Rb1, malus domestica, Malus sylvestris, malvidin, Manganese, Mannitol, Matairesinol, m-Cymene, Medicarpin, Medioresinol, Melatonin, menaquinone, Methoxybrassinin, Methyl salicylate, Methyleugenol, Methylisoeugenol, Monotropein, myricetin, myristic acid, Myristicin, Myristoleic acid, myrtenal, N,N-Dimethyltryptamine, N6-(delta2-Isopentenyl)adenine, N-Acetylhistamine, naringenin, Naringenin chalcone, naringin, Neobetanin, Neochlorogenic acid, neoeriocitrin, Neoglucobrassicin, neohesperidin, Neoxanthin, Nepetin, nerolidol, Nervonic acid, Nevadensin, N-feruloyl tyramine, N-Hexadecanoic acid, n-Hexane, Niacin, nicotinic acid, Nitrate, nobiletin, nomilin, Noradrenaline, nothofagin, Notoginsenoside A, Notoginsenoside C, Notoginsenoside D, Notoginsenoside Fa, Notoginsenoside Fe, Notoginsenoside K, notoginsenoside R1, Notoginsenoside R4, n-Pentadecane, o-Coumaric acid, Octacosan-1-ol, Octadecanoic acid, Octanoic acid, Oenin, Oenothein B, Oestrone, oleanolic acid, Oleic acid, Ononin, orientin, Orobanchyl acetate, ortho-Topolin, Osthol, oxalic acid, Oxypeucedanin, Palmitaldehyde, palmitic acid, Palmitoleic acid, Panax ginseng, Panax notoginseng saponins, Panaxydol, panaxynol, Panaxytriol, pantothenic acid, Patuletin, p-Coumaric acid, p-Coumaroyl CoA, p-Cymene, Pedunculagin, Pelargonidin, pelargonidin 3-galactoside, pelargonidin-3,5-diglucoside, pelargonidin-3-glucoside, Pelargonin, pentadecanoic acid, peonidin, petunidin, Phloretin, phloridzin, phlorizin, p-hydroxybenzoic acid, Phylloquinone, Phytoene, Phytofluene, Phytol, Pimaric acid, Pine bark, Pineapple, pinene, pinolenic acid, Pinoresinol, Pinosylvin, Pinosylvin methyl ether, pinus pinaster, Piper Nigrum, piperine, Polypodine B, Pomegraniin A, potassium, Pratensein, Pregnenolone, Primulin, proanthocyanidin B1, proanthocyanidin trimer, proanthocyanidins, Procurcumenol, Procyanidin A2, Procyanidin B1, Procyanidin B2, Procyanidin B3, Procyanidin B4, Procyanidin B5, Procyanidin C1, procyanidins, prodelphinidins, Progesterone, Progoitrin, proline, Prolycopene, Protocatechuic acid, Protodioscin, pterostilbene, Punica granatum, Punicalagin, Punicalin, Punicic acid, Punigluconin, Pycnogenol, Pyrocatechin, Pyruvic acid, Quercetin, Quercetin-3-O-glucoside, quercitrin, Quinic acid, Raffinose, Rebaudioside A, Rebaudioside C, reseveratrol, Resveratrol, Retinol, Rhamnetin, Rhamnocitrin, Rhodioloside, Ribitol, Riboflavin, Rosmarinic acid, Rubrofusarin, Rubusoside, rutin, rutoside, Sabine, sabinene, Saccharopine, Salicylic acid, Sandaracopimaric acid, Sanguiin H-6, Schaftoside, Schottenol, scopoletin, Secoisolariciresinol, selenium, Se-Methylselenocysteine, Shiitake, Shikimic acid, sinapic acid, Sinigrin, Sissotrin, Skatole, So-NCC-1, So-NCC-3, Sorbitol, spathulenol, spermine, Sphinganine, Sphondin, spinacetin, Spinach, Spinasterol, Spiraeoside, stearic acid, Stevia rebaudiana, Steviol, Steviol glycosides, steviolbioside, Stevioside, Stigmasterol, strawberry, Strictinin, Succinic acid, Succinic acid methyl ester, Sucrose, sulforaphane, Syringaldehyde, Syringaresinol, syringic acid, Tambulin, Tanegool, tannins, Tartaric acid, Taurine, Tauroursodeoxycholic acid, taxifolin, Tellimagrandin I, terpinen-4-ol, terpineol, Testosterone, Tetradecanoic acid, Tetraethyl orthosilicate, Theobromine, Theophylline, Thiamine, Thujopsene, Tiliroside, tocopherols, trans-Citral, trans-p-coumaryl alcohol, trans-Zeatin, trans-zeatin riboside, Trehalose, Triacontanol, Tricetin, Tricin, trilinolein, Tryptamine, turmerone, Tyramine, ubiquinone, ubiquinone-10, UDP-D-glucose, Umbelliferone, Undecanal, Uridine, Urolithin A, Urolithin B, Urolithin C, Ursolic acid, Vaccinium corymbosum, valoneic acid dilactone, Vanillic acid, Vanillin, Vicenin II, Vitamin A, vitamin B, vitamin B12, Vitamin B3, vitamin B9, vitamin C, vitamin D, vitamin D2, vitamin D3, Vitamin E, vitexin, Xanthyletin, zeaxanthin, Zedoarondiol, Zinc.

# CM Interactome

AAAS, ABCA1, ABCB1, ABCB4, ABCB6, ABCB8, ABCC2, ABCD1, ABCD3, ABCD4, ABCE1, ABCG8, ABI1, ABI2, ACLY, ACO2, ACOX1, ACSL1, ACSL3, ACSL4, ACTA2, ACVRL1, ADA, ADAM10, ADAM17, ADAMTS13, ADCK4, ADCYAP1, ADIPOQ, ADIPOR1, ADIPOR2, ADM, ADRB1, ADRB2, ADRBK1, AGO2, AGPS, AGT, AHR, AIP, AKAP5, AKR1C1, AKR1C2, AKT1, AKT2, ALK, ALKBH5, ALOX5, ALOX5AP, ALPL, AMN, AMPH, AMT, ANGPTL4, ANK2, ANXA1, ANXA2, AOX1, APAF1, APC, APEX1, APH1A, APLP1, APLP2, APOA1, APOB, APOE, APP, AQP1, AQP2, AQP3, AQP4, AQP5, AR, ARAF, AREG, ARF1, ARHGEF9, ARNT, ARRB2, ASIC1, ASIC2, ASIC3, ASPH, ATF2, ATF3, ATF4, ATF6, ATG12, ATG3, ATG4B, ATG5, ATG7, ATM, ATOX1, ATP1A3, ATP2A1, ATP2A2, ATP7A, ATP7B, ATR, ATRX, ATXN1, AURKA, AVP, AXL, B2M, BACE1, BACH1, BACH2, BAD, BAK1, BAP1, BAX, BCL2, BCL2L1, BCL2L11, BCR, BCS1L, BECN1, BGLAP, BGN, BID, BIN1, BIN2, BIRC5, BLM, BMP2, BMP4, BMP6, BMP7, BNIP3, BRAF, BRCA1, BRCA2, BRCC3, BRD4, BSG, BTC, BTK, BUB1, C2, C3, C3AR1, C4A, C5, C9, CA2, CA3, CA4, CA9, CABP1, CACNA1A, CACNA1B, CACNA1C, CACNA1D, CACNA1H, CACNA1S, CACNB2, CALB1, CAMK2A, CAMK2D, CAMK4, CAMP, CAPN1, CARD9, CARM1, CASK, CASP1, CASP3, CASP4, CASP7, CASP8, CASP9, CASQ2, CASR, CAST, CAT, CATSPER1, CAV1, CAV2, CBL, CBLB, CBLC, CCK, CCL11, CCL19, CCL2, CCL21, CCL3, CCL4, CCL5, CCL8, CCNB1, CCND1, CCR2, CCR4, CCR5, CCR7, CCS, CD14, CD163, CD19, CD1A, CD1D, CD22, CD274, CD320, CD34, CD38, CD4, CD40, CD44, CD47, CD5, CD59, CD63, CD74, CD81, CD86, CD9, CDC20, CDH1, CDH2, CDK1, CDK2, CDK6, CDK8, CDK9, CDKN1A, CDKN1B, CDKN2A, CDR1, CDR2, CEACAM1, CEBPA, CENPU, CETP, CFB, CFD, CFH, CFP, CFTR, CGA, CHD1, CHD3, CHD4, CHEK2, CHL1, CHRNA7, CHUK, CIC, CIITA, CIT, CITED2, CLDN1, CLDN16, CLDN19, CLDN2, CLDN4, CLDN5, CLOCK, CLP1, CLU, CNR1, COA6, COL11A1, COL1A1, COL1A2, COL2A1, COL3A1, COL4A1, COL4A3, COL4A4, COL4A5, COL7A1, COMMD1, COPA, COPE, COPRS, COPS5, COQ3, COQ4, COQ5, COQ6, COQ7, COQ9, COX11, COX17, COX19, CP, CPT1A, CR2, CRABP2, CREB1, CREBBP, CRH, CRP, CRY1, CRY2, CS, CSF1R, CSNK1A1, CST3, CTGF, CTLA4, CTNNB1, CTSB, CTSL, CUBN, CUL3, CX3CL1, CX3CR1, CXCL1, CXCL10, CXCL12, CXCL8, CXCL9, CXCR1, CXCR3, CXCR4, CYB5R3, CYBA, CYBB, CYLD, CYP17A1, CYP19A1, CYP1A1, CYP1A2, CYP1B1, CYP24A1, CYP26A1, CYP27B1, CYP2A6, CYP2B6, CYP2C19, CYP2C8, CYP2C9, CYP2E1, CYP2R1, CYP3A4, CYP4A11, CYP51A1, CYP7A1, DAB1, DAG1, DAXX, DBI, DCC, DCN, DDB1, DDIT3, DDT, DDX58, DES, DET1, DHFR, DIABLO, DISC1, DKK1, DLG4, DLK1, DMD, DNAJB1, DNMT1, DNMT3A, DNMT3B, DNMT3L, DPP4, DR1, DRD1, DRD2, DRD4, DSG1, DSG2, DSP, DTNB, DUOX2, DUSP1, E2F1, ECD, EDN1, EDNRB, EED, EEF2, EGF, EGFR, EHHADH, EHMT1, EHMT2, EIF2AK2, EIF2AK3, EIF4EBP1, ELAVL1, ELL, ELN, EMD, ENG, ENO1, EP300, EPHB2, EPO, EPOR, ERBB2, ERBB4, ERC1, ERCC1, ERCC6, EREG, ERG, ERN1, ESR2, EXO1, EZH2, F10, F11, F2, F3, F5, F7, F8, F9, FABP1, FABP4, FABP5, FADD, FANCD2, FAP, FASLG, FASN, FBN1, FBXL5, FBXO32, FECH, FEN1, FER, FES, FFAR4, FGF1, FGF19, FGF2, FGF21, FGF23, FGF8, FGFR1, FGFR2, FGFR3, FGFR4, FGR, FH, FHL1, FLT1, FLT3, FMR1, FN1, FOS, FOXA1, FOXO1, FOXO3, FOXO4, FOXP3, FST, FTH1, FTL, FTO, FUS, FUT2, FXYD1, FXYD2, FYN, G3BP1, G6PD, GABARAP, GABARAPL1, GABBR1, GABBR2, GABRA1, GABRA2, GABRA3, GABRA4, GABRA5, GABRA6, GABRB1, GABRB2, GABRB3, GABRG2, GAD1, GAD2, GADD45A, GAPDH, GAS6, GATA3, GBA, GCA, GCG, GCLC, GCLM, GCSH, GDA, GDF11, GDNF, GFAP, GFER, GGA1, GGA3, GH1, GHRH, GIP, GJA1, GJB2, GLDC, GLP1R, GLP2R, GLRA1, GLRA3, GLRB, GLUD1, GLUD2, GMPS, GNAI2, GNAS, GNB2L1, GP2, GPER1, GPHN, GPRC6A, GPX1, GPX3, GRIN1, GRIP1, GRK5, GRK6, GRM1, GRM5, GRN, GRP, GRPR, GSC, GSDMD, GSK3B, GSN, GSTM1, GSTP1, H2AFX, HAMP, HAP1, HBB, HBEGF, HCCS, HCFC1, HCK, HCRT, HDAC1, HDAC2, HDAC3, HDAC4, HDAC6, HDAC7, HEPH, HES1, HFE, HFE2, HGF, HGS, HIF1A, HK1, HK2, HLA-A, HLA-B, HLA-C, HLA-DPB1, HLA-DQA1, HLA-DQB1, HLA-DRB1, HMGB1, HMOX1, HNF1A, HNF4A, HNRNPA1, HNRNPL, HOXA9, HP, HPR, HPX, HRAS, HSCB, HSD17B10, HSF1, HSF2, HSP90AB1, HSPA1A, HSPA5, HSPA8, HSPA9, HSPB1, HTT, IAPP, IBSP, ICAM1, ICE1, ID1, IDE, IFNG, IGF1, IGF1R, IGF2, IGFBP1, IKBKB, IKBKE, IL10, IL17A, IL18, IL1A, IL1B, IL1RL1, IL2, IL22, IL2RA, IL3, IL33, IL4, IL5, IL9, ILK, INA, INO80, INS, INSR, IQGAP1, IRAK1, IREB2, IRF1, IRF3, IRS1, IRS2, ISCU, ISG15, ITCH, ITGA2B, ITGB1, ITM2B, ITPR1, ITPR2, ITPR3, JAG1, JAK1, JAK2, JAK3, JARID2, JPH2, JUN, JUND, KAT5, KDM2A, KDM4A, KDR, KIT, KL, KLF1, KLF4, KLF6, KLRK1, KMT2D, KRAS, L3MBTL1, LAMA4, LAMP1, LAMP2, LAT2, LBP, LCAT, LCN2, LDLR, LEP, LEPR, LGALS3, LGALS9, LGI1, LHB, LIF, LIG1, LMBRD1, LMNA, LOXL1, LOXL2, LPA, LPL, LRP1, LRRK2, LTB, LTF, LYN, M6PR, MADD, MAF, MAFB, MAGEL2, MAGT1, MAL, MALT1, MAP1LC3A, MAP1LC3B, MAP1LC3C, MAP2K1, MAP2K2, MAP2K3, MAP2K6, MAP3K1, MAP3K5, MAPK1, MAPK3, MAPT, MAT1A, MAT2A, MAVS, MAX, MB, MB21D1, MBD4, MC4R, MCL1, MCM6, MCM7, MCU, MDH2, MDM2, MECP2, MED25, MEF2C, MEF2D, MEIS1, MEN1, MET, METTL14, METTL3, MFN1, MFN2, MGP, MICA, MICU1, MIF, MIP, MITF, MLH1, MLKL, MMACHC, MMADHC, MMP1, MMP2, MMP3, MNT, MPC1, MPC2, MPG, MPL, MPO, MR1, MRE11A, MSH3, MSN, MST1, MSX2, MTA1, MTOR, MTR, MTRR, MUC1, MUC4, MUSK, MYB, MYC, MYCN, MYD88, MYH6, MYH7, MYOG, NANOG, NAPA, NAPB, NAPG, NBR1, NCAM1, NCL, NCOA1, NCOA4, NCOR1, NCOR2, NDUFB8, NDUFS2, NDUFS4, NEB, NEDD8, NEO1, NES, NF1, NFAT5, NFATC1, NFATC2, NFATC3, NFE2L2, NFKB1, NFKBIA, NFS1, NFU1, NGF, NKX2-1, NLRC4, NLRP1, NLRP3, NMB, NME1, NMT2, NOD2, NOS1, NOS3, NOTCH1, NOTCH4, NOX1, NOX4, NPHS2, NPM1, NPPA, NPPB, NPR1, NPR3, NPSR1, NPY, NQO1, NR1H3, NR1I2, NR3C1, NR3C2, NR4A1, NRAS, NRF1, NRG1, NSD1, NSF, NTRK2, NUP98, OCLN, ODC1, OGG1, OLIG2, OLR1, OPRM1, ORAI1, ORAI2, ORAI3, ORC1, OSM, OXT, OXTR, P2RX2, P2RX3, P2RY12, P2RY2, PADI4, PAF1, PAK1, PARG, PARK2, PARK7, PARP1, PAX3, PAX6, PBRM1, PC, PCBP1, PCBP2, PCCA, PCCB, PCNA, PCSK9, PDCD1, PDE4A, PDE4D, PDGFRA, PDIA3, PDPK1, PDPN, PDSS1, PDSS2, PDZK1, PELO, PELP1, PER1, PER2, PEX14, PEX3, PEX5, PF4, PFKFB3, PFKP, PGD, PGF, PGK1, PGR, PGRMC1, PHB, PHIP, PHLPP1, PI3, PIAS4, PIF1, PIK3CA, PIM1, PIN1, PINK1, PIP, PIR, PITX2, PKD1, PKD2, PLA2G4A, PLAA, PLAT, PLCB1, PLCB2, PLD2, PLG, PLIN2, PLIN3, PLIN5, PLK1, PLN, PLTP, PMAIP1, PNPLA2, PNPLA3, POLE, POMC, PON1, POR, POSTN, POU5F1, PPARA, PPARD, PPARG, PPARGC1A, PPM1A, PRC1, PRDX1, PRDX2, PRDX6, PRKCA, PRKCD, PRKCE, PRKCQ, PRL, PRMT1, PRMT5, PRMT8, PRNP, PROM1, PRX, PSEN1, PSEN2, PSENEN, PTBP1, PTEN, PTGER1, PTGER4, PTGES, PTGIR, PTGS1, PTGS2, PTH, PTH1R, PTHLH, PTPN1, PTPN11, PTPN22, PTPN6, PTPRC, PTTG1, PTX3, PVR, PXN, RAB11A, RAB3A, RAC1, RAD17, RAD50, RAD51, RAD52, RAD54L, RAG1, RAG2, RAMP1, RAN, RAP1A, RAP1B, RAPGEF4, RARA, RARB, RB1, RBCK1, RBP1, RBP4, RBPJ, RDX, RECQL4, REL, RELA, RELB, RELN, REN, REPIN1, RET, REV1, RFC1, RFWD2, RGL2, RHEB, RHO, RHOA, RIPK3, ROCK1, ROCK2, RORA, RORC, RPA3, RPN1, RPS2, RTN3, RTN4, RUNX1, RUNX2, RUNX3, RUVBL1, RUVBL2, RXRA, RYR1, RYR2, RYR3, S100A1, S100A10, S100A2, S100A4, S100A6, S100A7, S100A8, S100A9, S100B, SAG, SARM1, SAT1, SATB2, SCARB1, SCARB2, SCGN, SCLY, SCN10A, SCN1A, SCO1, SCO2, SCT, SDC4, SDHA, SDHAF1, SDHB, SDHC, SDHD, SELE, SELENBP1, SELK, SELP, SENP1, SERPINE1, SET, SETD2, SETD7, SFN, SFTPC, SH2D1A, SHBG, SHC1, SHMT2, SIRT1, SIRT2, SIRT3, SIRT4, SIRT6, SKI, SKP1, SKP2, SLC11A2, SLC17A7, SLC18A3, SLC1A1, SLC1A2, SLC26A3, SLC2A4, SLC32A1, SLC34A1, SLC3A2, SLC40A1, SLC4A4, SLC5A1, SLC6A2, SLC6A3, SLC6A4, SLC7A10, SLC7A11, SLC7A7, SLC7A8, SLC7A9, SLC9A1, SLC9A3, SLN, SMAD1, SMAD2, SMAD3, SMAD4, SMAD5, SMAD7, SMARCA2, SMARCA4, SMARCB1, SMC2, SMC3, SMO, SMS, SNAI1, SNAP25, SNAPIN, SNCA, SOCS1, SOCS3, SOD1, SOD2, SOD3, SORL1, SOS1, SOS2, SOST, SOX2, SOX9, SP1, SP7, SPARC, SPHK1, SPHK2, SPN, SPP1, SPRY2, SPTB, SQSTM1, SRC, SRCAP, SREBF1, SREBF2, SRF, SRI, SRR, SST, SSTR2, STAC3, STAR, STAT1, STAT3, STAT5A, STAT6, STIM1, STIM2, STK11, STRA6, STRAP, STUB1, STX1A, STX2, SUMO1, SUMO2, SUMO3, SUZ12, SV2A, SYK, SYN1, SYP, SYT1, SYT2, SYVN1, TAC1, TAGLN, TANK, TAP1, TAP2, TARDBP, TBK1, TBP, TBX3, TCEB1, TCF4, TCF7L1, TCN2, TDG, TEK, TET1, TET2, TET3, TF, TFAM, TFAP2A, TFEB, TFR2, TFRC, TGFB1, TGFBR1, TGM2, TH, THRB, TIGIT, TIMP1, TJP1, TLR2, TLR4, TLR7, TLR8, TLR9, TMBIM6, TMEM173, TMPRSS6, TNC, TNF, TNFRSF10A, TNFRSF11A, TNFRSF11B, TNFRSF13B, TNFRSF13C, TNFRSF17, TNFRSF1A, TNFSF10, TNFSF11, TNFSF13, TNFSF13B, TNNT2, TOP2A, TOR1A, TP53, TP53BP1, TP63, TPM1, TPR, TRAF2, TRAF4, TRAF6, TRDN, TREM2, TRIB1, TRIO, TRPA1, TRPC1, TRPC3, TRPC4, TRPC5, TRPC6, TRPC7, TRPM3, TRPM4, TRPM6, TRPM7, TRPV1, TRPV3, TRPV4, TRPV5, TRPV6, TSC1, TSHR, TSPO, TTR, TUBB3, TUSC3, TXNIP, TXNRD2, TYMS, UBC, UBE2D1, UBE2D3, UBE3A, UBIAD1, UCHL1, UCN, UGT1A1, UGT1A10, UGT1A3, UGT1A8, UGT1A9, UGT2B7, UHRF1, ULK1, UNC13A, UQCRC2, USF2, USP7, VAMP2, VASP, VAV1, VCL, VDAC1, VDR, VEGFA, VIM, VIMP, VPS35, VWF, WLS, WNT1, WNT2, WNT3A, WRN, WWP1, XBP1, XIAP, XPA, XPC, YAP1, ZAP70, ZEB2.

.

# Supplementary Table 1

| PROTEIN NETWORK OVERLAP ANALYSIS OF CM INTERACTOME WITH DISEASE NETWORKS | | | | |
| --- | --- | --- | --- | --- |
| **Term Description** | **Observed** | **Background** | **Strength** | **False Discovery Rate** |
| Bone resorption disease | 10 | 12 | 0.91 | 0.00026 |
| Hyperglycemia | 9 | 10 | 0.95 | 0.00046 |
| Coenzyme q10 deficiency disease | 9 | 11 | 0.9 | 0.00074 |
| Rickets | 9 | 11 | 0.9 | 0.00074 |
| Osteoporosis | 9 | 11 | 0.9 | 0.00074 |

1. Khalaf, D., Krüger, M., Wehland, M., Infanger, M. and Grimm, D., 2019. The effects of oral l-arginine and l-citrulline supplementation on blood pressure. Nutrients, 11(7), p.1679. [↑](#endnote-ref-1)
2. Kim, D.H., Meza, C.A., Clarke, H., Kim, J.S. and Hickner, R.C., 2020. Vitamin D and endothelial function. Nutrients, 12(2), p.575. [↑](#endnote-ref-2)
3. Mahdi, A., Kövamees, O. and Pernow, J., 2020. Improvement in endothelial function in cardiovascular disease-Is arginase the target?. International journal of cardiology, 301, pp.207-214. [↑](#endnote-ref-3)
4. Stanhewicz, A.E. and Kenney, W.L., 2017. Role of folic acid in nitric oxide bioavailability and vascular endothelial function. Nutrition reviews, 75(1), pp.61-70. [↑](#endnote-ref-4)
